# NPS MedicineWise application in supporting medication adherence in chronic heart failure: an acceptability and feasibility pilot study

**DOI:** 10.3389/fdgth.2023.1274355

**Published:** 2023-11-15

**Authors:** Jessica Chapman-Goetz, Nerida Packham, Kitty Yu, Genevieve Gabb, Cassandra Potts, Adaire Prosser, Margaret A. Arstall, Christine Burdeniuk, Alicia Chan, Teena Wilson, Elizabeth Hotham, Vijayaprakash Suppiah

**Affiliations:** ^1^ Clinical and Health Sciences, University of South Australia, Adelaide, SA, Australia; ^2^ Consumer Medicines Information Services, NPS MedicineWise, Surry Hills, NSW, Australia; ^3^ e-Health, NPS MedicineWise, Melbourne, VIC, Australia; ^4^ Department of Cardiology, Noarlunga GP Plus Super Clinic, Adelaide, SA, Australia; ^5^ SA Pharmacy, Flinders Medical Centre, Bedford Park, SA, Australia; ^6^ Department of Cardiology, Northern Adelaide Local Health Network, Adelaide, SA, Australia; ^7^ Department of Cardiology, Flinders Medical Centre, Bedford Park, SA, Australia; ^8^ Department of Cardiology, Royal Adelaide Hospital, Adelaide, SA, Australia; ^9^ Integrated Cardiovascular Clinical Network, Country Health South Australia, Adelaide, SA, Australia; ^10^ Australian Centre for Precision Health, University of South Australia, Adelaide, SA, Australia

**Keywords:** mobile health (mHealth), mobile phone application, heart failure, medication adherence, mobile technology

## Abstract

**Introduction:**

Heart failure (HF) is an increasing global concern. Despite evidence-based pharmacotherapy, associated morbidity and mortality remain high. This study aimed to assess the acceptability, feasibility, and value of the NPS MedicineWise dose reminder app in a tiered, pharmacist-led intervention to address medication non-adherence in patients with HF.

**Methods:**

This prospective, single-blinded, randomised controlled trial recruited 55 patients with HF between September 2019 and October 2020. Participants were randomly assigned to either the intervention or control arms. Intervention participants used the app which prompted medication administration at each dosing interval. Control participants received standard care and remained blinded to the app throughout the study. Treatment non-adherence prompted a tiered, pharmacist-led intervention. Comparison of the Self-Efficacy for Appropriate Medication Use Scale (SEAMS) at baseline and 6-months measured the app's value in supporting medication adherence. Secondary outcome measures included self-reported medication knowledge, health-related quality of life, psychological wellbeing, and signs and symptoms of HF. Data were analysed using standard statistical tests with significance set at *α* 0.05.

**Results:**

Approximately half of respondents reported managing HF and medications better by using the MedicineWise app (Tier 1). Most respondents expressed satisfaction with the in-app messages (Tier 2) and pharmacists' phone calls (Tier 3). The intervention participants demonstrated a significant improvement in the SEAMS between baseline and 6-months follow-up.

**Discussion:**

It is feasible and potentially of value to use the MedicineWise app with a tiered, pharmacist-led intervention to support medication adherence in patients with HF. Our findings provide clinicians with “real-world” information on the practicality and potential value of using mobile health to support treatment adherence in patients with HF.

**Trial registration number:**

Australian New Zealand Clinical Trials Registry Clinical trial registration number: ACTRN12619000289112p (http://www.ANZCTR.org.au/ACTRN12619000289112p.aspx)

## Introduction

The prevalence of heart failure (HF) in Australia has been estimated to be around 1%–2%, rising significantly to 10% in those aged 75 years and above (1, 2). Significant morbidity in this population contributes to frequent and prolonged hospitalizations with approximately four-fifths being hospitalized at least once and up to three-quarters dying within five years of diagnosis (1, 3).

Pharmacotherapy is the foundation of HF management with evidence supporting improvement in morbidity and mortality (4–6). Despite implementing polypharmacy as strongly advocated by several guidelines (4, 5, 7), clinical outcomes remain suboptimal (8).

As therapeutic efficacy is dependent on routine medication administration, non-adherence has been shown to increase both frequency of hospitalizations and relative risk of all-cause mortality (8). Medication non-adherence in HF can be further complicated by co-morbidities like dementia and depression, disease, progression and severity, and inherent regimen complexity and high “pill burden” (9–12). Studies have shown that approximately one-fifth to one-third of HF patients may not consistently take their medications as prescribed (13, 14).

The exponential growth in mobile/smartphone ownership has driven interest in mobile health (mHealth) as a strategy to address medication non-adherence (15). Relative ease of use, accessibility and low-cost of mobile devices make mHealth a particularly desirable modality for patient-centered services (15–17). Since the emergence of smartphones, mHealth has evolved from telephone call and text message-based supports to include applications (apps), social media platforms and health information systems (18, 19). These can better promote interventions that support health-related behaviors including medication adherence via dose reminders/alerts, dose tracking, prescription refill reminders and/or storing medication-related information (20, 21).

The NPS MedicineWise dose reminder app is a free medicine and health management app funded by the Australian government to help patients and their carers, keep track of their medicines and other important health information with the aim, among others, of improving medication adherence, being a source of reliable medical information and storing patients' health and medicine information in one place (22, 23).

The primary aim of this pilot study was to determine the acceptability, feasibility, and value of incorporating the NPS MedicineWise dose reminder app in a tiered, pharmacist-led intervention to address medication non-adherence in patients with HF. The Self-Efficacy for Appropriate Medication Use Scale (SEAMS) (24) will be used to determine the app's value in supporting medication adherence. The secondary aim was to determine the impact of the app on signs and symptoms of HF and quality of life at 6-months.

## Materials and methods

### Study design and population

This was a prospective, single-blinded observational randomized controlled trial. The full study protocol has been published elsewhere (25). Australian New Zealand Clinical Trials Registry Clinical trial number: ACTRN12619000289112p (http://www.ANZCTR.org.au/ACTRN12619000289112p.aspx). Briefly, the study was conducted at six South Australian investigator centers between September 2019 and October 2020. Recruitment of study participants took place from 1st November 2019 to 30th April 2020 and the last patients were followed till October 2020. Rural participants were recruited through the Integrated Cardiovascular Clinical Network (ICCNet) and interstate participants via an NPS MedicineWise social media advertisement. Eligible participants were briefed about the study by the treating physician or cardiology nurse either in person or by phone during routine clinic visits or, for rural patients, during their scheduled tele-health consultation/ appointments. Potential participants were provided with a study summary and referred to the study team for eligibility screening (Supplementary Table S1) prior to a preliminary interview - face-to-face, at any participating hospital, or via phone. At this initial visit, written informed consent was obtained from those who agreed to participate, and a baseline assessment was completed. Interstate participants reached via the NPS social media advertisement were directed to an eligibility survey. Upon its completion, a preliminary interview occurred as above. Written informed consent was obtained via post or email and a baseline assessment was completed. The eligibility criteria for this study were broadly designed (Supplementary Table S1) to include older individuals and those with comorbidities like depression as they can be underrepresented in mHealth research (9, 26).

Sample size calculation for this study was based upon a medium (*d* = 0.5) to large (*d* = 0.8) effect size as measured by Cohen's d (27). While the upper limit of real-world adherence to cardiovascular medications was estimated to be 60% (28), a patient is considered to have good adherence when ≥80% of doses had been taken (29). To detect an increase in adherence from 60% to 80%, a minimum total sample size of 52 participants was needed to provide 80% power, with a one tail 0.05 significance level with an effect size of 0.7 (30). To allow for dropouts, a total of 55 participants will be recruited for this study as previously described (25).

Participants were randomly allocated to intervention or control. Control participants did not have access to any medication reminder apps (including the MedicineWise app) and remained blinded to the existence of an intervention arm for study duration. This was facilitated by using a different patient information sheet and consent form for the recruitment of control and intervention arm participants. Participation in the control arm involved regular follow ups of functional assessments (questionnaires), medication adherence and knowledge questionnaire and the collection of laboratory results. This was intended to minimize recognized limitations of earlier research where blinding was not present (19). However, researchers undertaking data collection were not blinded due to the personalized and tiered nature of the intervention.

At baseline, intervention participants were instructed in using the MedicineWise app. Following set-up, a reminder (Tier 1) prompted medication administration at each dosing interval. If non-adherence was suggested from 24 h reports (critical medications) or 72 h reports (non-critical medications), the participant was escalated through a tiered, pharmacist-led intervention which consisted of in-app messages (Tier 2) and phone calls from the NPS MedicineWise Medicines Line Pharmacists (Tier 3). Critical medications were those that form the foundation of pharmacotherapy due to established morbidity or mortality benefits (5). Medicines Line is a national consumer medicines information telephone service, delivered by NPS MedicineWise. The NPS MedicineWise Medicines Line (Medicines Line) – was a “real world” national consumer medicine information service delivered by pharmacists and operated between Mondays to Fridays 9 am to 5 pm AEST (excluding New South Wales public holidays). The Medicines Line service and the MedicineWise app were funded by federal Department of Health and Aged Care to 31 December 2022.

The study protocol was approved by the respective institutional Human Research Ethics Committees (Protocol numbers R20190302 and 202450).

### Outcome measure

The primary outcome was the acceptability, feasibility, and value of this approach in supporting medication adherence. Comparison of the SEAMS (24) at baseline and 6-months was used to measure the app's value in supporting medication adherence. Secondary outcome measures included health-related quality of life, psychological wellbeing, and signs and symptoms of HF.

### Data collection and statistical analysis

Trial data were collected via face-to-face or phone interviews as determined by participant preference, geographical location, or local governmental health guidelines for COVID-19 infection control. Baseline data were collected at the time of recruitment and repeated at each follow-up with details of any changes to participants' medication recorded. Part A of the Self-care of Heart Failure Index (SCHFI) questionnaire (27) was utilized to determine whether control participants had used any drug administration aids, medication reminder apps or other electronic reminder systems for daily medication administration, since the previous interview. The satisfaction survey was completed with intervention participants upon study completion by another researcher not associated with data collection.

Continuous variables were presented as the mean ± standard deviation (SD), or the median and interquartile range (IQR) for data that were not normally distributed (skewed). Categorical (or discrete) variables were expressed as the number and percentage of frequency. Thematic analysis was performed on qualitative data to generate emerging and overarching themes (31, 32).

The paired t-test was used within each arm to examine continuous data collected at baseline and 6-month follow-up. Differences in categorical variables were assessed using the McNemar test. Between group analysis was conducted with the two-sample t-test (continuous variables) or the *χ*^2^ test (categorical variables). The Wilcoxon Signed Rank test or Mann–Whitney *U* test were used for continuous data that did not display a normal distribution. Analysis of the SEAMS, medication adherence and knowledge, EQ-5D-5l (33), Short Form 36 Health Survey version 2 (SF-36v2) 34, Depression Anxiety and Stress Scales (DASS-21) (35) and SCHFI (36) questionnaires were performed with SPSS software, with statistical significance set at *α* 0.05.

## Results

**Figure 1:**
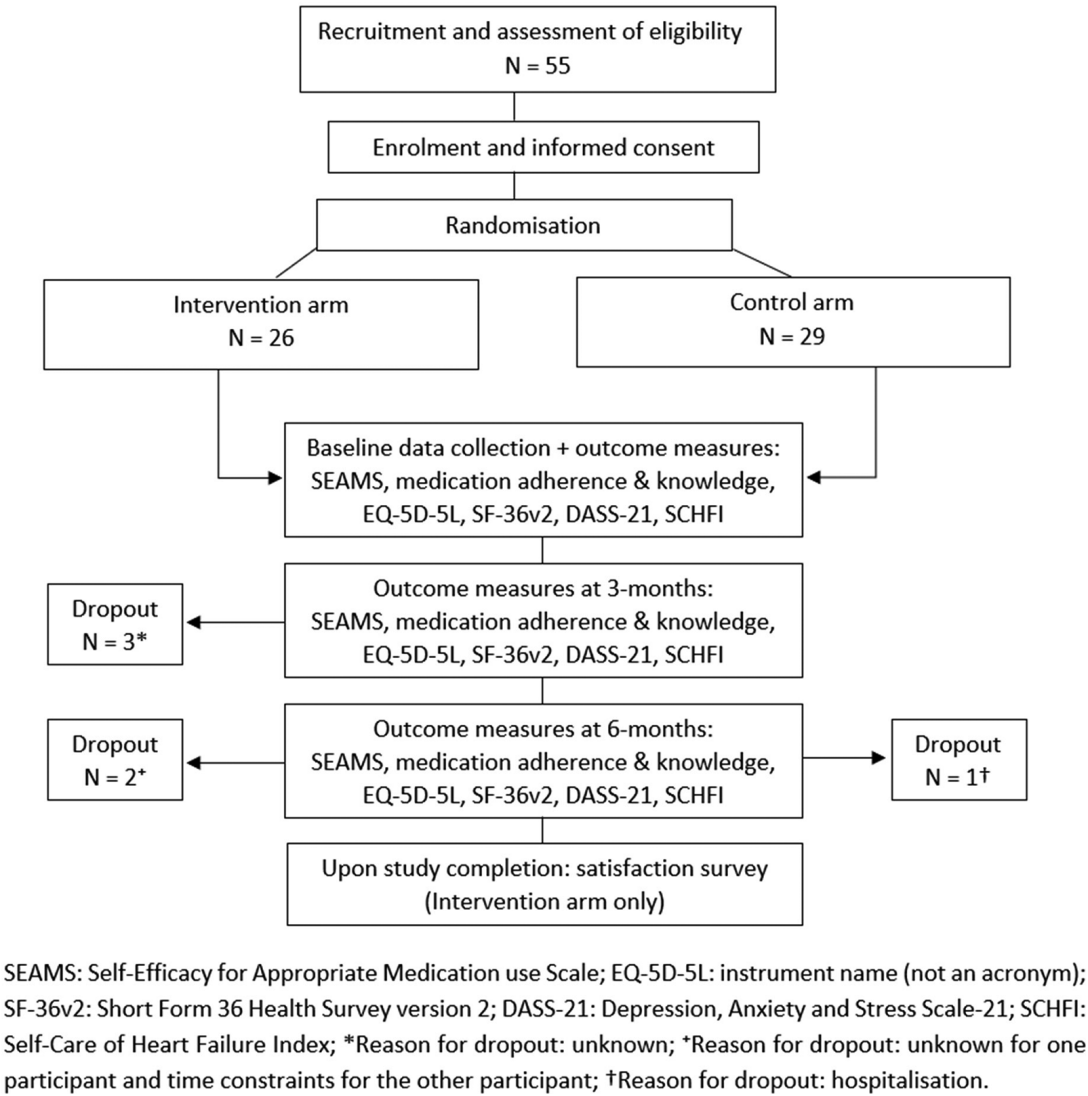
Schematic diagram of trial design with representation of sample size and loss follow-up (dropout).

Of the referred 80 patients with HF, 55 agreed to participate in the study (Figure 1). Time constraints and changing social circumstances (such as moving into an aged care facility or interstate) were reasons for declining to participate. Forty-nine individuals (89%) were followed until study completion (Figure 1). Three intervention arm participants were lost by the 3-month and one by 6-month follow-ups, without explanation. Time constraints prompted one intervention arm participant to withdraw prior to the last interview and hospitalization prevented one control arm participant from undergoing her 6-month follow-up.

The study cohort characteristics are presented as a whole cohort as there were no statistically significant differences between the two groups (Table 1). While participants were predominantly Caucasian (95%) and male (67%), a near equal number lived either with a partner (married or de facto) or alone (divorced, widowed or single). The mean age and body mass index (BMI) were 64 years (SD: 13 years) and 33 kg/m^2^ (SD: 8 kg/m^2^), respectively. Most participants (65%) were unemployed or retired and had no regular daytime activity. Only seven individuals (13%) in the cohort identified as current smokers. Commonly documented comorbidities included type 2 diabetes mellitus (38%), hypertension (35%), atrial fibrillation (22%) and cardiovascular disease (20%). Only five individuals (9%) had a history of depression. Nearly all participants (85%) were classified as having either “no” symptoms of HF or limitations in ordinary physical activity [NYHA class I (42%)], or “mild” symptoms of HF including fatigue and shortness of breath with ordinary physical activity [NYHA class II (43%)]. On average, participants took 4 (± 1) “critical” and 3 (± 3) “non-critical” medications (Supplementary Table S2) which were mostly administered from an organizer such as a dosette or Webster® pack (58%).

**Table 1:** Demographic characteristics of participants.

Characteristics	Total (*n* = 55)	Control (*n* = 29)	Intervention (*n* = 26)	*P* value^a^
Ethnicity, (%)				
Caucasian	52 (95)	28 (97)	24 (92)	0.488
Non-Caucasian	3 (5)	1 (3)	2 (8)	
Gender, (%)				
Male	37 (67)	18 (62)	19 (73)	0.385
Female	18 (33)	11 (38)	7 (27)	
Marital status, (%)				
Married/Defacto	27 (49)	16 (55)	11 (42)	0.341
Divorced or widowed or single	28 (51)	13 (45)	15 (58)	
Age, y, mean (SD)	64 (13)	68 (13)	60 (11)	0.511
Body mass index (kg/m^2^), mean (SD)	33 (8.1)	33 (8.4)	33 (8)	0.911
Daytime activity, (%)				
No regular daytime activity	36 (65)	23 (79)	13 (50)	0.07
Employed	19 (35)	6 (21)	13 (50)	
Smoking status, (%)				
Current smoker	7 (13)	3 (10)	4 (15)	0.851
Ex-smoker	28 (51)	15 (52)	13 (50)	
Non-smoker	20 (36)	11 (38)	9 (35)	
Comorbidities, (%)				
Type 2 diabetes mellitus	21 (38)	11 (38)	10 (38)	0.983
Hypertension	19 (35)	10 (34)	9 (34)	
Atrial fibrillation	12 (22)	6 (21)	6 (23)	
Cardiovascular disease	11 (20)	5 (17)	6 (23)	
Depression	5 (9)	2 (8)	3 (11)	
NYHA class I-III, (%)				
Class I	23 (42)	7 (24)	16 (61)	0.25
Class II	24 (43)	17 (59)	7 (27)	0.148
Class III	8 (15)	5 (17)	3 (12)	0.102
Class I or II	47 (85)	24 (83)	23 (88)	0.741
Number of critical medications, mean (SD)^2^	4 (1)	4 (1)	4 (2)	0.581
Number of non-critical medications, mean (SD)^b^	3 (3)	4 (4)	3 (3)	0.24
Medication management system, (%)				
Original containers	23 (42)	9 (31)	14 (54)	0.077
Medication organiser (such as a dosette or Webster® pack)	32 (58)	20 (69)	12 (46)	

^a^ Comparison of control vs. intervention arm as calculated by the *χ*^2^ test or the two-sample -Test

^b^ As listed in Supplementary Table S2.

### Tier one: app reminders

A total of 2,679 “missed” doses were logged in the app's database between December 2019 and October 2020 (Table 2). However, 779 (29%) of these were incorrectly recorded as “missed”. Investigation by the NPS MedicineWise team revealed that the automated “batch-sync” had intermittently malfunctioned between March-April 2020 and not all data were transferring to the cloud within 24 h. A series of “hotfixes” was released in June, July, and September 2020 to assist in diagnosing, monitoring and subsequent resolution of the malfunction. In addition, functional improvements to the app were implemented via the last “hotfix,” allowing users to visualize successful recording of their “TAKEN” doses in the cloud as a time stamp on their device. Despite the “batch-sync” difficulty, a total of 1,900 (71%) “missed” doses were correctly recorded over the study.

**Table 2:** True and false “missed” doses from tier 1 interventions.

		True interventions^a^	False interventions^b^
Month – Year	Participants who received Tier 1 interventions (*n*)	Tier 1 “missed” doses (*n*)	Tier 1 “missed” doses (*n*)
Dec-19	5	33	3
Jan-20	7	34	9
Feb-20	9	20	9
Mar-20	18	92	53
Apr-20	24	205	96
May-20	24	312	116
Jun-20	24	296	84
Jul-20	19	429	101
Aug-20	17	297	120
Sep-20	14	106	133
Oct-20	4	76	55
Total		1,900	779

^a^ True “missed” doses pertain to those that were correctly recorded within the app's database. The term implies that participants were non-adherent with tapping “TAKEN” in the app, though they may have been adherent with their medications.

^b^ False “missed” doses pertain to those that were incorrectly recorded within the app's database due to the “batch-sync” malfunction.

### Tier two: push notification messages

A total of 749 “push” notification messages were sent between December 2019 and October 2020 (Table 3). Of these, 426 (57%) were in response to true (correctly recorded) “missed” doses. The remainder were erroneously sent due to the false (incorrectly recorded) “missed” doses incurred by the “batch-sync” malfunction. This malfunction only affected participants using Android devices (*n* = 20).

**Table 3:** True and false tier 2 “push” notification messages.

		True interventions^a^	False interventions^b^
Month – Year	Participants who received Tier 2 interventions (*n*)	Tier 2 “push” notification messages (*n*)	Tier 2 “push” notification messages (*n*)
Dec-19	5	11	3
Jan-20	7	25	7
Feb-20	9	18	9
Mar-20	18	60	44
Apr-20	24	67	48
May-20	24	35	50
Jun-20	24	65	34
Jul-20	19	72	31
Aug-20	17	52	23
Sep-20	14	15	44
Oct-20	4	6	30
Total		426	323

^a^ True interventions pertain to Tier 2 “push” notification messages that were generated in response to correctly recorded “missed” doses within the app's database.

^b^ False interventions pertain to Tier 2 “push” notification messages that were generated in response to “missed” doses that were incorrectly recorded within the app's database due to the “batch-sync” malfunction.

### Tier 3: contact from an NPS MedicineWise medicines line pharmacist

Over the study period, 17 (71%) intervention arm participants were escalated to Tier 3 and received on average 3 ± 2 phone calls from a Medicines Line pharmacist. The remaining 7 (29%) participants did not require a Tier 3 intervention for the entire duration of the study.

Due to the unforeseen technical issues like the “batch-sync” malfunction the nominated cap of 3 phone calls per participant was removed. In all, a total of 96 Tier 3 interventions were conducted between December 2019 and October 2020. However, no instances of medication non-adherence were identified. The main issues were technical difficulties with the MedicineWise app (39%) and non-adherence with the app itself (43%). Common explanations for the loss of engagement included: hospitalization, temporary cessation of medication (primarily prior to surgery), inconsistent smartphone use and delay in downloading the app onto an upgraded device. Accidentally entering a new medication (furosemide) as daily, rather than ‘when required,’ prompted intervention on another 2 occasions (2%) and contact could not be made for the remaining 16 (17%) Tier 3 interventions.

### Primary objectives: acceptability, feasibility, and value of the app

Only half of the initial 26 participants in the intervention arm took part in the satisfaction survey. Although all respondents felt “very” or “somewhat” confident in their ability to use the MedicineWise app after initial training, about half (54%) stated that they “would not have been confident” to use the app without the training. Although there were other app functions available, only 22% had used the app to record blood pressure, weight, and the contact details of their medical specialists.

Over the study period, the app prompted medication administration “all of the time” for 5 (38%) respondents, “some of the time” for another 5 (38%) and “none of the time” for the rest 3 (24%). Only 3 (24%) individuals felt that these reminders were “useful”. Most respondents 9 (69%) tapped the “TAKEN” icon in less than 15 min while the rest did so within 15 to 30 min after taking their medication. The reassurance provided by tapping “TAKEN” was deemed to be “very useful” by 4 (31%) respondents. Three (24%) respondents did not find tapping “TAKEN” useful as visual evidence of dose administration was already provided by their medication organizer.

Nine (90%) of the ten individuals who received Tier 2 interventions expressed satisfaction with the messages and did not suggest any improvement. Tier 3 interventions were received by 8 (62%) respondents, of whom, five (62.5%) considered the conversation with the Medicines Line pharmacists to be “very helpful” or “good”. Most respondents (75%) felt “very comfortable” in asking these pharmacists additional medicine-related questions. The rest (25%) felt that this was “not needed” as they already had good relationships with existing healthcare providers. Three participants (23%) were not contacted by a Medicines Line pharmacist for the entire duration of the study.

The MedicineWise app helped 6 (46%) respondents to manage their HF and medications by acting as a prompt or additional reminder. Of the 7 (54%) respondents who did not feel that the app helped them, 2 (29%) explained that they would have taken their medications regardless. Five (38%) respondents stated that they were “very likely” to continue using the app as they found it helpful and well-integrated into their daily routines. Of the reminder, 2 (15%) said “unlikely” to continue using the app without further elaboration and the rest (46%) chose not to answer that question. Nevertheless, approximately half of respondents (54%) would recommend the app to other patients with HF.

Self-efficacy with medication adherence was subjectively measured with the SEAMS (24). Both control and intervention arm participants had high self-reported medication adherence across all time points (Supplementary Table S5). While there was no difference between groups at baseline or 6-months follow-up, the intervention arm had a marginally significant (p = 0.049) increase in the median SEAMS score over this period (Table 4).

**Table 4:** Median SEAMS scores for the control and intervention arms at baseline, 3- and 6-months follow-up.

	MEDIAN SEAMS SCORE (IQR)	*P*-value^a^	*P*-value^b^
CONTROL			
Baseline (*n* = 29)	46 (4)	Baseline between groups = 0.133	Baseline vs. 6 months in control = 0.535
3-months follow-up (*n* = 29)	47 (4)		
6-months follow-up (*n* = 28)	46.5 (5)		
INTERVENTION			
Baseline (*n* = 26)	44.5 (6)	6 months between groups = 0.671	Baseline vs. 6 months in intervention = 0.049
3-months follow-up (*n* = 23)	44 (4)		
6-months follow-up (*n* = 21)	47 (4)		

^a^ Comparison of control and intervention arms as calculated by the Mann–Whitney *U* test^.^

^b^ Comparison of baseline and 6-months follow-up within each arm as calculated by the Wilcoxon Signed Rank test.

### Secondary outcome measures

Even though nearly all control and intervention arm participants (93% vs. 88% respectively, p = 0.549) believed that their medications were improving their health, most participants (79% vs. 85% respectively, *p* = 0.61) were able to recall a time when they did not take all prescribed medication doses. Recurring themes were forgetfulness, running out of medication, unpleasant side effects (including dizziness or frequent urination from diuretics), “pill burden”, distraction, and loss of routine.

Previous research has shown that individuals with a chronic health condition may become “non-adherent” with medication after 6-months of treatment (37, 38). When asked if this scenario applied, significantly fewer control than intervention arm participants (17% vs. 42% respectively, *p* = 0.041) confirmed that it did. Whilst most participants (90% vs. 96% respectively, *p* = 0.354) stated that they would tell their health professionals if non-adherent, approximately half (52% vs. 58% respectively, *p* = 0.657) reported never being asked if they were taking or using their medications. Due to the infrequent or sporadic nature of non-adherence, 14 (48%) control and 9 (35%) intervention arm participants considered that volunteering this information to their health professional was irrelevant.

Although only 52% of control and 62% of intervention participants knew the names of all of their medications at baseline (*p* = 0.463), most (83% vs. 96% respectively, *p* = 0.111) participants could visually identify them (Supplementary Table S3 and S4) at baseline, without change at 6 months. Knowledge of actual or potential drug interactions was lacking for participants in both arms at baseline and 6-months follow-up (Supplementary Table S3 and S4). While the knowledge deficit at each interval was more prominent for controls, the difference between groups was not significant.

Apart from a significant improvement in pain or discomfort at 6 months in the control arm (*p* = 0.039), all the other aspects of health domains tested in the EQ-5D-5l were not significant within and between the groups at the three time points. Results from the SF-36v2 indicating control arm participants experienced significantly less physical health (*p* = 0.003) and emotional health (*p* = 0.006) problems by 6-months follow-up is concordant with their substantially greater limitations at baseline when compared with intervention arm participants (Supplementary Table S5 and S6). Further, both measures were not significantly different by 6 months for the participants in the intervention arm. Additionally, unlike control arm participants, the intervention arm had a statistically significant (*p* = 0.032) improvement in the median score of part A of the SCHFI between baseline and 6 months (Supplementary Table S7). Emotional states measured by the DASS-21 and parts B and C of the SCHFI showed no significant changes in either group by the end of the study period.

## Discussion

This is the first study to assess the acceptability, feasibility, and value of the MedicineWise app by extending the app's capabilities by including a tiered, pharmacist-led intervention to address medication non-adherence in patients with HF. Our findings suggest that this approach was acceptable to HF patients. Although delivery of Tier 1 intervention was feasible due to automation, Tiers 2 and 3 interventions required more input from the Medicines Line pharmacists' team. An improvement in the SEAMS between baseline and 6-months follow-up in the intervention participants shows the potential value of the app in supporting medication adherence of these patients. However, the secondary outcome measures remained unchanged for the duration of the study.

As shown in previous studies, it is possible that with adequate training, older patients can successfully incorporate technology into their daily lives (17, 26, 39, 40). Even though the app training was well received by the majority (77%), about half (54%) of survey respondents stated that they would have lacked confidence to use the app without training. The subsequent ease with which the MedicineWise app was integrated into the participants' daily lives showed that an older population can be adequately trained to use mHealth apps and to receive meaningful outcomes while achieving satisfaction from ongoing engagement (26, 39).

The alarm reminders of Tier 1 intervention acted as a safeguard against known contributors to non-adherence, including distraction and forgetfulness. Park et al. (2019) (20) similarly concluded that mHealth apps could overcome these barriers and support medication-taking behavior in older individuals with cardiovascular disease. In this study, tapping “TAKEN” provided visual reassurance for some participants that medication had been administered, which eased reliance upon memory and concern of potential non-adherence. However, as shown by Andre et al. (2019) (16), these prompts lost their usefulness in participants who have already established a daily routine for taking their medications.

Intervention participants who “missed” a dose of a critical medication for 24 h or a non-critical medication for 3 days received a Tier 2 “push” notification message within the MedicineWise app. Even though, 43% of the total 749 Tier 2 messages were sent because of technical issues, most survey respondents (90%) who received the messages were satisfied and did not suggest further improvement. This is consistent with prior research where text message-based interventions to support medication adherence were well received by patients with chronic diseases (41). Reassuringly, none of the 96 Tier 3 interventions identified medication non-adherence. Instead, non-adherence with the app was responsible for 41 (43%) of these phone calls. Telephone follow-ups have been successfully used by clinicians, particularly nurses, to support community-dwelling patients with chronic diseases (20, 42, 43).

Intervention participants, who received the tiered intervention, confirmed the value of the Medicine Line pharmacists when surveyed. They demonstrated an improvement in SEAMS-measured medication adherence between baseline and 6-months, in contrast to control participants who did not demonstrate a similar change, reinforcing the usefulness of health professional involvement as previously reported.

Most of the secondary measures remained unchanged across the study. While this study did not feature an educational component to improve health literacy, neither group was better able to name or visually identify their medications, verify that correct medications were supplied, or state understanding of actual or potential drug interactions, when compared to baseline. This suggests that clinicians may need to utilize a multi-modal approach including individualized education to improve patients' medication knowledge as suggested by Sheilini et al. (2019) (44). The intervention did not have a significant effect on health-related “quality of life” across timepoints. Depression scores were maintained within the “normal” range, although reduction in median scores was greater for the intervention group. However, this was not observed in the other two domains of the DASS-21. Up to a third of patients with HF experience depression and even more exhibit depressive symptoms (45). Either the prevalence of mental illness in this cohort was too low to demonstrate a significant change or improved medication adherence alone may not be sufficient to improve emotional states of depression, anxiety, or stress in patients with HF.

The MedicineWise app assumes that medications had been administered when the “TAKEN” icon was tapped. This may have caused reporting biases as intervention-arm participants could have acted in accordance with socially desired behavior by tapping “TAKEN” even if doses of medication were missed. The reverse might also have been true whereby medications were administered but the “TAKEN” icon not tapped. The possible discordance between medication administration and tapping “TAKEN” was a limitation when interpreting study results and determining implications for clinical practice. These assumptions are a recognized drawback of objective adherence assessment methods (46). However, this was mitigated if the participant was escalated to a Tier 3 intervention because a Medicines Line pharmacist could ascertain reasons for app non-adherence (e.g., forgetfulness or technical difficulties) and confirm whether the patient was taking their medications.

Inclusion of participants with multiple device models, different operating systems, and in particular older device models and operating systems, may have contributed to the technical issues observed during the study. The rapid change in smartphone technology “forces” app development teams to use “digital languages” that are compatible with both older and newer device models and operating systems. This has been highlighted to be a common challenge in the consumer app space contributing to high user attrition rates and unreliable engagement with mHealth (11, 12). Technical issues encountered during this study highlight the vital importance of ongoing app maintenance and user support. The Medicines Line pharmacists provided a valuable link between participants and Consumer Support to resolve app-related issues, by a series of updates, “hotfixes,” and troubleshooting provided by the app development team, Consumer Support service and the research team.

The COVID-19 pandemic posed a significant recruitment challenge for this study. Prior to the implementation of local health guidelines for COVID-19 infection control, study participants were referred from HF clinics and exercise classes. With the conversion of the former to telehealth and the closure of the latter, patients with HF were comparatively “out of sight.” As clinicians reported an increase in workload from changes in healthcare delivery, the challenges of usual roles and added responsibilities likely impeded their capacity to identify and refer potentially eligible patients with HF, impacting on participant recruitment.

The relatively small sample size may have resulted in potential Type II errors and limited the strength of these findings. Ideally, replicating this study with a larger sample size could strengthen the power of between group analyses to detect changes in secondary outcome measures. Extending the follow-up period beyond 6-months may provide added insight into longer-term acceptability, feasibility, and value of the tiered intervention in supporting medication adherence in patients with HF.

Additionally, interventions that support medication-taking behavior may prove more beneficial for patients with a prior history of non-adherence. As there was already a high level of adherence in this cohort, most of the secondary aims remained unchanged. Future research could add the criterion of including only those with a history of non-adherence. However, non-adherence is notoriously difficult to quantify and the feasibility of busy clinicians being able to screen and refer patients must be considered.

## Conclusions

The rise in smartphone ownership has piqued interest in mHealth as a strategy to address medication non-adherence. While evidence supports its use, mHealth alone may not be the solution to such a complex problem. Although the use of mHealth and mobile phone app became more critical during the pandemic lockdowns and restrictions, they have an established value to aiding patients with all aspects of their health and medications (47). Mobile phone apps are another tool in a community pharmacy environment where pharmaceutical care has become a broader, more demanding, and holistic endeavor (48). The value of mobile phone apps could be enhanced within a more complex, structured program as shown previously (49) and here. They would be more effective with strong pharmacist support, but this support should be balanced with workload and perceived financial disadvantages (50).

A major finding of this study was that the utilization of the MedicineWise app in a tiered, pharmacist-led intervention was acceptable to patients with HF. However, initial training was necessary to enhance confidence in navigating the app, particularly for anyone with lower baseline technology proficiency. Nevertheless, technical issues could limit ongoing app engagement without access to Consumer Support services. Tier 1 interventions were easily delivered, but Tiers 2 and 3 interventions, although valuable, required additional resources. In conclusion, this pilot study provides evidence that incorporating a smartphone medication reminder app into a tiered intervention in a real-world situation is acceptable, feasible, and is able to support the medication adherence of patients with HF.

## Data Availability

The raw data supporting the conclusions of this article will be made available by the authors, without undue reservations.
