# Supplementary material for: NPS MedicineWise application in supporting medication adherence in chronic heart failure: an acceptability and feasibility pilot study
Source: Front Digit Health. 2023 Nov 15;5:1274355. doi: 10.3389/fdgth.2023.1274355 (PMC10684918; doi:10.3389/fdgth.2023.1274355)
Supplement: Supplementary file 1 [file Table1.docx]

**SUPPLEMENTARY MATERIAL**

**NPS MedicineWise application in supporting medication adherence in chronic heart failure: An acceptability and feasibility pilot study**

Jessica Chapman-Goetz, Nerida Packham, Kitty Yu, Genevieve Gabb, Cassandra Potts, Adaire Prosser, Margaret A Arstall, Christine Burdeniuk, Alicia Chan, Teena Wilson, Elizabeth Hotham, Vijayaprakash Suppiah*

*Correspondence: Vijayaprakash Suppiah

Postal address: UniSA Clinical and Health Sciences, City West Campus, University of South Australia, Adelaide, SA 5000, Australia

Email: vijay.suppiah@unisa.edu.au

Tel: +61 8 8302 1130

S1 Table. Eligibility criteria

S2 Table. Critical and non-critical medication list

S3 Table. Medication knowledge at baseline.

S4 Table. Medication knowledge at 6-months follow-up.

S5 Table. Median RP scores for the control and intervention arms at baseline, 3- and 6-months follow-up.

S6 Table. Median RE scores for the control and intervention arms at baseline, 3- and 6-months follow-up.

S7 Table. SCHFI part A: Median scores for the control and intervention arms at baseline, 3- and 6-months follow-up.

**S1 Table. Eligibility criteria**

| Inclusion criteria | Exclusion criteria |
| --- | --- |
| Age 18 years or older | Palliative heart failure |
| Systolic heart failure ^+^ | NYHA functional class IV |
| NYHA functional class I-III for ≥ 3-months | Malignancy or diastolic heart failure |
| LVEF < 50% * | Life expectancy ≤ 6-months |
| Stable or stabilised condition | Use of other medication reminder apps ** |
| Participant or carer with access to a smartphone | Unable to read or speak English |
| Able to receive/respond to emails and phone calls | No access to a smartphone or email |

^+^ as confirmed on echocardiography, NYHA: New York Heart Association, LVEF: Left ventricular ejection fraction, * at the time of HF diagnosis as per the 2018 Australian HF guidelines, ** or other electronic reminder systems for daily medication administration.

**S2 Table. Critical and non-critical medication list**

| **Class** | **Active ingredients** | **Critical vs non-critical** | **Comment** |
| --- | --- | --- | --- |
| ACE inhibitors | Captopril | Critical | Include all single AND combination products containing these active ingredients  Include both salts of perindopril (erbumine and arginine) |
|  | Enalapril +/- hydrochlorothiazide +/- lercanidipine |  |  |
|  | Fosinopril +/- hydrochlorothiazide |  |  |
|  | Lisinopril |  |  |
|  | Perindopril arginine +/- amlodipine +/- indapamide |  |  |
|  | Perindopril erbumine +/- indapamide |  |  |
|  | Quinapril +/- hydrochlorothiazide |  |  |
|  | Ramipril +/- felodipine |  |  |
|  | Trandolapril +/- verapamil |  |  |
| Angiotensin II receptor blockers | Candesartan +/- hydrochlorothiazide | Critical | Include all single AND combination products containing these active ingredients |
|  | Eprosartan +/- hydrochlorothiazide |  |  |
|  | Irbesartan +/- hydrochlorothiazide |  |  |
|  | Losartan |  |  |
|  | Olmesartan +/- amlodipine +/- hydrochlorothiazide |  |  |
|  | Telmisartan +/- amlodipine +/- hydrochlorothiazide |  |  |
|  | Valsartan +/- amlodipine +/- hydrochlorothiazide |  |  |
| Beta blockers | Bisoprolol | Critical |  |
|  | Carvedilol |  |  |
|  | Metoprolol succinate |  |  |
|  | Metoprolol tartrate |  |  |
|  | Nebivolol |  |  |
| Loop diuretics | Frusemide | Critical |  |
|  | Bumetanide |  |  |
|  | Ethacrynic acid |  |  |
| Aldosterone antagonists | Spironolactone | Critical |  |
|  | Eplerenone |  |  |
| Anti-arrhythmics | Digoxin | Critical |  |
|  | Amiodarone | Critical |  |
| Cardio-tonic agent | Ivabradine | Critical |  |
| Angiotensin receptor / neprilysin inhibitor | Sacubitril/Valsartan | Critical |  |
| Nitrates | Isosorbide mononitrate | Critical |  |
|  | Isosorbide dinitrate | Critical |  |
| Arteriolar vasodilator | Hydralazine | Critical |  |
| Anticoagulants | Warfarin | Critical |  |
|  | Apixaban |  |  |
|  | Rivaroxaban |  |  |
|  | Dabigatran |  |  |
| Sodium-glucose co-transporter 2 inhibitor | Empaglifozin | Non-critical |  |
| Calcium channel blockers | Amlodipine | Non-critical | Include all single AND combination products containing these active ingredients |
|  | Felodipine | Non-critical |  |
| Thiazide or thiazide-like diuretics | Indapamide | Non-critical | Include all single AND combination products containing these active ingredients |
|  | Hydrochlorothiazide | Non-critical |  |
| Iron | Ferrous sulphate +/- ascorbic acid +/- folic acid | Non-critical | Brand names: Ferrogradumet, Ferro Liquid, Ferrograd C, FGF, Fefol |
|  | Ferrous fumarate +/- folic acid | Non-critical | Brand names: Ferro-tab, Ferro-F |
|  | Iron polymaltose | Non-critical | Brand name: Maltofer |
|  | Iron Sucrose | Non-critical | Brand name: Venofer |
|  | Ferrous fumerate/folic acid/ascorbic acid/cyanocobalamin | Non-critical | Brand name: Ferro-sachets, Iron melts |
| Lipid lowering | Atorvastatin +/- amlodipine +/- ezetimibe | Non-critical | Include all single AND combination products containing these active ingredients |
|  | Simvastatin +/- ezetimibe |  |  |
|  | Fluvastatin |  |  |
|  | Pravastatin |  |  |
|  | Rosuvastatin +/- ezetimibe |  |  |
|  | Fenofibrate |  |  |
|  | Ezetimibe |  |  |

**S3 Table. Medication knowledge at baseline.**

| BASELINE  MEDICATION KNOWLEDGE | Total  (n = 55) | Control  (n = 29) | Intervention  (n = 26) | P value^a^ |
| --- | --- | --- | --- | --- |
| Do you know all the names of your medicines?  No, n (%)  Yes, n (%) | 24 (44)  31 (56) | 14 (48)  15 (52) | 10 (38)  16 (62) | 0.463 |
| Can you visually identify all your medicines?  No, n (%)  Yes, n (%) | 6 (11)  49 (89) | 5 (17)  24 (83) | 1 (4)  25 (96) | 0.111 |
| Do you always make sure that you get the right medicines every time you fill a prescription?  No, n (%)  Yes, n (%) | 4 (7)  51 (93) | 1 (3)  28 (97) | 3 (12)  23 (88) | 0.248 |
| Are you aware of any interactions between the medicines that you are on?  No, n (%)  Yes, n (%) | 34 (62)  21 (38) | 16 (55)  13 (45) | 18 (69)  8 (31) | 0.284 |

^a^ Comparison of control vs intervention arm as calculated by the Chi-square test

**S4 Table. Medication knowledge at 6-months follow-up.**

| MEDICATION KNOWLEDGE AT  6-MONTHS FOLLOW-UP | Total  (n = 49) | Control  (n = 28) | Intervention  (n = 21) | P value^a^ |
| --- | --- | --- | --- | --- |
| Do you know all the names of your medicines?  No, n (%)  Yes, n (%) | 22 (45)  27 (55) | 14 (50)  14 (50) | 8 (38)  13 (62) | 0.407 |
| Can you visually identify all your medicines?  No, n (%)  Yes, n (%) | 8 (16)  41 (84) | 5 (18)  23 (82) | 3 (14)  18 (86) | 0.737 |
| Do you always make sure that you get the right medicines every time you fill a prescription?  No, n (%)  Yes, n (%) | 2 (4)  47 (96) | 2 (7)  26 (93) | -  21 (100) | 0.211 |
| Are you aware of any interactions between the medicines that you are on?  No, n (%)  Yes, n (%) | 33 (67)  16 (33) | 18 (64)  10 (36) | 15 (71)  6 (29) | 0.597 |

^a^ Comparison of control vs intervention arm as calculated by the Chi-square test

**S5 Table. Median RP scores for the control and intervention arms at baseline, 3- and 6-months follow-up.**

|  | **RP**  **MEAN SCORE (SD)** | **P value^a^** | **P value^b^** |
| --- | --- | --- | --- |
| **CONTROL** | | |  |
| Baseline (n = 29) | 45.5 (27) | **0.003** | **0.03**  -  0.278 |
| 3-months follow-up (n = 29) | 55.4 (30.8) |  |  |
| 6-months follow-up (n = 28) | 60.3 (33.3) |  |  |
| **INTERVENTION** | | |  |
| Baseline (n = 26) | 63 (30.7) | 0.149 | **0.03**  -  0.278 |
| 3-months follow-up (n = 23) | 65.8 (33.1) |  |  |
| 6-months follow-up (n = 21) | 70.2 (30.1) |  |  |

^a^ Comparison of baseline and 6-months follow-up within each arm as calculated by the paired t-Test

^b^ Between group comparison of baseline and 6-months follow-up as calculated by the two-sample t-Test

**S6 Table. Median RE scores for the control and intervention arms at baseline, 3- and 6-months follow-up.**

|  | **RE**  **MEAN SCORE (SD)** | **P value^a^** | **P value^b^** |
| --- | --- | --- | --- |
| **CONTROL** | | |  |
| Baseline (n = 29) | 69 (33) | **0.006** | 0.543  -  0.692 |
| 3-months follow-up (n = 29) | 79.3 (28.2) |  |  |
| 6-months follow-up (n = 28) | 83.6 (21.8) |  |  |
| **INTERVENTION** | | |  |
| Baseline (n = 26) | 74 (28.6) | 0.138 | 0.543  -  0.692 |
| 3-months follow-up (n = 23) | 86.2 (19.7) |  |  |
| 6-months follow-up (n = 21) | 86.1 (21.5) |  |  |

^a^ Comparison of baseline and 6-months follow-up within each arm as calculated by the paired t-Test

^b^ Between group comparison of baseline and 6-months follow-up as calculated by the two-sample t-Test

**S7 Table. SCHFI part A: Median scores for the control and intervention arms at baseline, 3- and 6-months follow-up.**

|  | **SCHFI PART A**  **MEAN SCORE (SD)** | **P value^a^** |
| --- | --- | --- |
| **CONTROL** | | |
| Baseline (n = 29) | 60.7 (13.7) | 0.363 |
| 3-months follow-up (n = 29) | 61.1 (15.9) |  |
| 6-months follow-up (n = 28) | 63.1 (12.8) |  |
| **INTERVENTION** | | |
| Baseline (n = 26) | 57.8 (12.5) | **0.032** |
| 3-months follow-up (n = 23) | 59.4 (12.7) |  |
| 6-months follow-up (n = 21) | 62.7 (14.1) |  |

^a^ Comparison of baseline and 6-months follow-up as calculated by the paired t-Test
